# Biomaterial Properties and Differentiation Strategies for Tenogenic Differentiation of Mesenchymal Stem Cells

**DOI:** 10.3390/cells14060452

**Published:** 2025-03-18

**Authors:** Brendon Roets, Heidi Abrahamse, Anine Crous

**Affiliations:** Laser Research Centre, Faculty of Health Science, University of Johannesburg, Doornfontein, P.O. Box 17011, Johannesburg 2028, South Africa; broets@uj.ac.za (B.R.); habrahamse@uj.ac.za (H.A.)

**Keywords:** tendon, tenocytes, tendinopathy, tenogenic differentiation, growth factors, biomaterials, tendon engineering

## Abstract

Tendinopathy is a prevalent musculoskeletal condition that affects both aging populations and individuals involved in repetitive, high-intensity activities, such as athletes. Current treatment options primarily address symptom management or involve surgery, which carries a significant risk of complications and re-injury. This highlights the need for regenerative medicine approaches that combine stem cells, biomaterials, and growth factors. However, achieving effective tenogenic differentiation remains challenging due to the absence of standardized differentiation protocols. Consequently, a review of existing research has been conducted to identify optimal biomaterial properties and growth factor protocols. Findings suggest that the ideal biomaterial for tenogenic differentiation should feature a 3D structure to preserve tenogenic expression, incorporate a combination of aligned micro- and nanofibers to promote differentiation, and require further investigation into optimal stiffness. Additionally, growth factor protocols should include an induction phase to initiate tenogenic lineage commitment, followed by a maintenance phase to support matrix production and maturation.

## 1. Introduction

Tendons are connective tissue structures that facilitate the transfer of forces generated by muscle to bone, allowing for efficient movement and stability within the musculoskeletal system [1]. Tendon injuries are some of the most common disorders within the musculoskeletal system and significantly impact an individual’s quality of life [2]. Tendons are prone to injury due to repetitive stress, trauma, and degenerative changes associated with aging [3]. According to the literature, the rotator cuff, Achilles tendon, and patellar tendon are the most frequently affected sites [4]. Tendon damage can be divided into acute damage, a sudden disruption in a normal tendon, or chronic damage, associated with degenerative changes [5]. Furthermore, tendons have a limited healing capacity due to their hypocellular and hypovascular nature and risk for healing complications [6]. The current treatment options include rest, physiotherapy, pharmacological drugs, and surgery. These options often result in ineffective repair of tendons to a pre-damaged state and a propensity for re-injury, highlighting the need for novel tissue regenerative approaches [7]. Mesenchymal stem cell (MSC) therapy has emerged as a promising therapeutic option for tendon regeneration due to the unique properties of these cells, including self-renewal and multilineage differentiation potential. MSCs can differentiate into various cell types, including tenocytes [8,9]. These are the main cell population within tendons responsible for tendon maintenance and repair. However, to harness the full therapeutic potential of MSCs, it is of critical importance to optimise differentiation strategies of MSCs to efficiently direct their differentiation towards the tenogenic lineage. Thus, this requires a deeper understanding of the interplay between the cellular microenvironment and signalling cues. This has prompted research to investigate biomaterials and growth factors to enhance the tenogenic differentiation of MSCs for the treatment of tendinopathy.

This review article aims to (1) provide a background on the structure and function of tendons, tendon healing, tendinopathy, and briefly discuss the current challenges associated with tendon repair; (2) highlight the molecular pathways regulating tenogenic differentiation; (3) summarise ideal biomaterial characteristics required for tendon regeneration; (4) discuss the use of growth factors in tenogenic differentiation protocols; and (5) lastly conclude with the ideal biomaterial characteristics for tendon engineering and growth factor usage in differentiation protocols and provide a prospective for future studies.

### 1.1. Tendon Structure and Function

**Figure 1:**
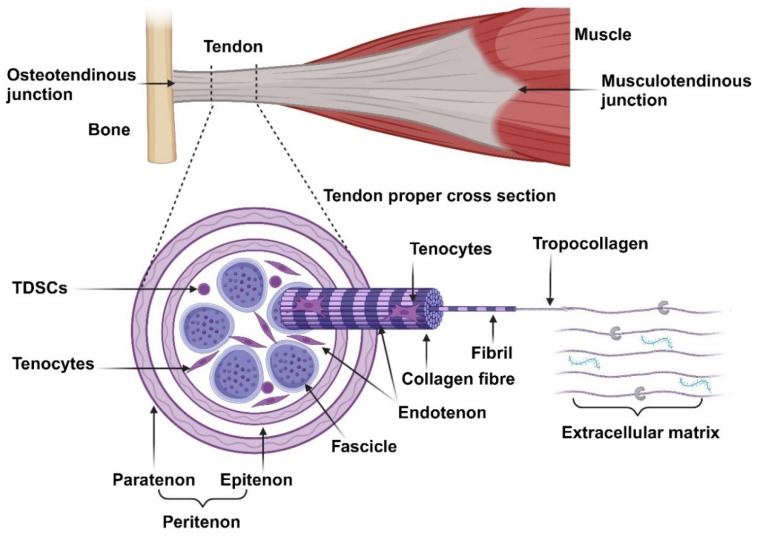
Overview of the anatomical structures of tendons. Tendons are organised into several hierarchical structures: fascicles, collagen fibres, fibrils, tropocollagen, and collagen molecules. The main cell population within tendons comprises of tenocytes, with a small subpopulation of TDSCs. These cells maintain the extracellular matrix, consisting of GAGs, PGs, and glycoproteins.

Broadly, a tendon can be divided into three regions, namely, the osteotendinous junction, tendon proper, and musculotendinous junction, as illustrated in Figure 1. The osteotendinous junction is the intersection between osseous and tendon tissue. Similarly, the musculotendinous junction is the intersection between muscle and tendon tissue. The tendon proper is the tendon tissue residing in the middle of the two insertion sites [10]. The gross anatomical structure of the tendon proper is organised in a hierarchical fashion and compartmentalised. The peritenon (extrinsic compartment) is a collection of loose connective tissue sheaths surrounding the peripheral surface of a tendon and is innervated by blood vessels and nerves. It consists of two layers, namely, the outer paratenon (tendon sheath) and inner epitenon (sub-tendon sheath) [11]. The tendon core (intrinsic compartment) is composed of a collection of fascicles, each consisting of numerous collagen fibres bound together and surrounded by the endotenon. This interfascicular matrix (IFM) is continuous with the epitenon and carries blood vessels and nerves deeper into the tendon structure. Each collagen fibre is composed of numerous fibrils consisting of collagen molecules [12]. The collagen molecules form a triple helix structure, known as tropocollagen, and are aligned on the tissue’s longitudinal axis [13]. This complex structure generates high tensile force and resilience under mechanical strength.

Tendons are composed of roughly 70% water and 30% dry mass. The dry mass consists mostly of collagen (65–80%) and a small amount of elastin (1–2%). The collagen resists tension and provides strength to the tendon while the elastic fibres allow the collagen to return to its resting state after tension is removed [14,15]. Collagen type I forms the main collagen component and structure within tendons (95%). Collagen III is the second most abundant collagen subtype and is mostly localised in the IFM. Furthermore, it is the first collagen to be produced during the healing phase of tendon repair, later replaced by collagen type I during the remodelling phase, and is also upregulated in tendinopathy. Type V collagen is found in the centre of collagen type I fibrils, serving as a template for fibrillogenesis. Traces of other collagen subtypes are also present within tendons [5,16].

The collagen molecules are embedded in an extracellular matrix (ECM) composed of glycosaminoglycans, proteoglycans, and glycoproteins. Proteoglycans (PGs) (decorin and biglycan) assist in fibrillogenesis by regulating the diameter, alignment, and spacing of the collagen fibrils [11]. Glycosaminoglycans (GAGs) have a high water-binding capacity and can form bridges between proteoglycan proteins, thereby contributing to the viscoelastic properties of tendons by maintaining the hydration levels of tendons, providing lubrication, and allowing tendons to possibly absorb and disseminate mechanical energy between adjacent collagen fibrils during loading [16]. Glycoproteins (Tenascin-C, thrombospondin, and thrombomodulin) regulates cell–matrix interactions. Tenascin-C (TNC) is produced in response to mechanical loading and serves as a ligand for integrins and modulates adhesion, participating in collagen fibre alignment and orientation [7]. Thrombospondin-4 mediates cell-to-ECM interactions and controls the deposition of ECM and collagen fibril organisation [17]. Thrombomodulin (Tnmd) promotes tenocyte proliferation and tendon maturation [11].

The resident cell populations within tendons are poorly defined due to a lack of tenogenic specific markers [18]. The main differentiation markers (scleraxis and tenomodulin), as well as the ECM markers (collagen and biglycan), used to characterise tenocytes are also expressed in various other connective tissues, albeit in different concentrations [19,20]. Tenocytes, terminally differentiated tendon-specific fibroblasts and the dominant cell population (roughly 95% of tendon cells) within tendons, are involved in synthesising and maintaining the ECM components [16]. These cells are spindle-shaped, reside between the collagen fibrils in longitudinal rows, and communicate via gap junctions [21]. Tendon-derived stem cells (TDSCs) are tendon-specific MSCs and maintain the tenocyte population. These cells are primarily localised to the epitenon and endotenon. They are spindle-shaped but more round compared to tenocytes and more metabolically active cells [21].

### 1.2. Tendon Healing

As previously reviewed the repair process within tendons occurs within three stages, namely, the inflammatory, proliferative, and remodelling phases [22]. During the inflammatory phase, vascular permeability increases, allowing inflammatory cells (neutrophils and macrophages) to enter the damaged tendon and secrete cytokines and growth factors (occurring over several days). The proliferative phase is associated with new blood vessel formation, the proliferation of tenocytes, and production of extracellular matrix proteins, especially collagen III (weeks). The remodelling phase is characterised by the replacement of collagen III with collagen I, the alignment of collagen to stress lines, and a reduction in tissue cellularity (months to years) [23]. Tendon healing can occur either via an intrinsic or extrinsic mechanism, depending on the mechanism of injury. Minor injuries with an intact epitenon result in intrinsic healing, mainly directed by cells of the tendon parenchyma, result in healing with superior mechanical properties and fewer complications [24]. In contrast, major injuries (disrupting the epitenon) result in extrinsic healing, directed by cells from the surrounding tissues, and are frequently associated with complications [25].

### 1.3. Tendinopathy and Associated Risk Factors

Tendinopathy is an umbrella term encompassing several tendon-related disorders characterised by pain, swelling, and reduced functionality [26]. Broadly, tendinopathy can be subdivided into tendinosis and tendinitis. Tendinosis is associated with non-inflammatory degenerative changes in response to the chronic overuse of tendon tissue [27]. Degenerative features include increased ground substance, disruption in the collagen-I-to-collagen-III ratio, the loss of collagen orientation with increased haphazard neovascularisation, and abnormal nerve innervation [28]. These changes weaken the tendon and pre-dispose it to tearing. In contrast, tendinitis refers to tendon inflammation resulting from micro-tissue tears that form when the tendon is acutely overloaded with either high or rapid tensile forces [29]. Uncertainty remains regarding whether these are two separate mechanisms or constitute one process occurring at different time points. Tendinopathy occurs as a result of frequent tendon injuries occurring during tendon healing, resulting in unsuccessful and disorganised repair [27].

Several intrinsic and extrinsic risk factors for tendinopathy have been identified and reviewed. Increased age predisposes one to tendinopathy as it is associated with degenerative changes such as increased matrix metalloproteinase production (disrupting the balance between ECM production and destruction) and a reduction in the healing capacity of tendons (a decreased vascular supply of nutrients) [6,30]. In one study, lower limb tendinopathy was more frequently identified in male runners compared to females and positively associated with an increase in the number of years participants participated in running activities [31]. Genetics influence tendon strength and repair capacity as evidenced by a high correlation between siblings and tendinopathy [30,32]. Predisposition has been associated with gene polymorphisms in *collagen* (*COL1A2, COL27A1, and COL5A1*) and *TNC* genes [33,34,35]. Tendinopathy was further associated with an increased body mass index, increasing the stain experienced by tendons [36]. Metabolic syndrome is associated with advanced glycation end products, dyslipidaemia, and fat deposition, predisposing one to tendinopathy through the generation of a pro-inflammatory environment, altering collagen structure and function, and interfering with tendon repair [37]. Athletes are commonly afflicted with tendinopathy, especially as a result of strenuous and repetitive activities, resulting in the overloading and chronic overuse of the tissue [38]. These risk factors (advancing age, high BMI, metabolic syndrome, and sports injuries) are frequently encountered in everyday life, and as such, the incidence of tendinopathy is expected to increase in the coming years.

### 1.4. Current Challenges Associated with Tendon Repair and Engineering

By nature, tendons are relatively hypovascular and hypocellular with a low metabolic rate. The combination of these characteristics prolongs the duration of the repair process due to the reduced delivery of nutrients and oxygen, as well as due to having a smaller cell population to effect the repairs [39,40]. Chronic low-grade inflammation, frequently encountered in tendinopathy, impairs the healing process within tendons [41]. Innate tendon healing is often accompanied by scar tissue formation and the deposition of disorganised ECM rather than the regeneration of native tendon tissue [23,42]. Although scar tissue provides temporary stability, it lacks the permanent structural integrity and mechanical properties of normal tendon tissue. Furthermore, scar tissue and surgical complications can also cause adhesions to form between tendons and the surrounding tissues. The net effect is impaired function, a failure to repair tendons to a pre-damaged state, and a high risk for re-injury [43]. Conventional treatment options are limited and do not provide satisfactory results in 25–45% of cases [44]. Non-surgical approaches, including rest, physical therapy, and pharmacological drugs, focus on managing symptoms, promote healing, and improve function rather than addressing the underlaying structural damage [45], thus highlighting the need for a functional regenerative medicine approach.

Significant progress has been made in the field of tendon tissue engineering. However, there are still several challenges associated with tendon repair. A lack of standardised tenogenic differentiation protocols and differentiation markers limits the comparison between tissue engineering research studies and prolongs the translation into clinical studies [46]. Effective tendon regeneration requires a sufficient number of cells to repair the damaged tissue. However, tenocytes have a limited capacity for proliferation and rapidly undergo lineage drift during in vitro expansion [47,48]. The poor survival and integration of in vitro expanded and differentiated cells into native tissue is a common problem in tissue regeneration [49]. Tendons have an extremely organised and complex ECM. A major hurdle in tendon engineering is guiding tendon tissue regeneration to replicate the organised ECM structure [50]. Several approaches have managed to produce the ECM, but the mechanical properties are frequently insufficient when compared to native tendon tissue. A further problem to clinical application is regarding how to optimise and upscale engineering strategies to clinically relevant sizes while simultaneously reducing the high cost associated with tissue engineering [51]. The tendon structure, tissue properties, and tenogenic phenotype change with respect to the tendon proper and integration sites, creating particular problems in effective tendon engineering at these interfaces, and will likely require different regeneration approaches [52].

## 2. Molecular Mechanisms Regulating Tenogenic Differentiation

Tenogenic differentiation is initiated by the expression of scleraxis (Scx), Mohawk (Mkx), and early growth response 1 (Egr1) transcription factors and maturation is characterised by the expression of tenomodulin (Tnmd) and ECM proteins (Col I, Col III, TNC, DCN, Thbs-4, and Biglycan) [53]. The tenogenic phenotype is regulated by a triad of biochemical stimulation (growth factors and chemical inducers), biophysical stimulation (biomaterial topography and stiffness), and mechanical stimulation (tensile loading) [47].

### 2.1. SMAD Signalling Pathway

**Figure 2:**
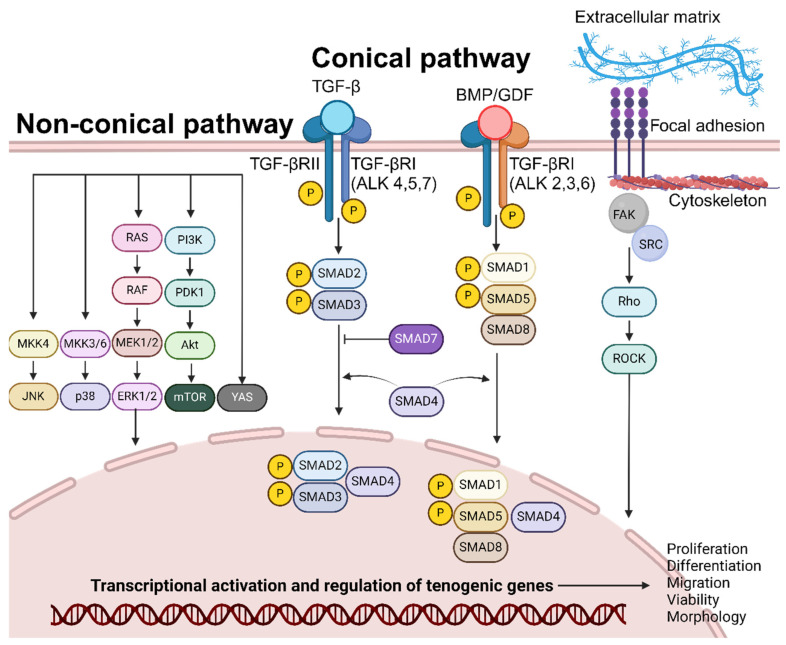
Overview of the molecular pathways regulating tenogenic differentiation. Tenogenic differentiation, simulated by growth factors (TGF, BMP, CTGF, ascorbic acid, bFGF, and PDGF) or substrate topography, is mediated by signalling cascades within the conical and non-conical pathways. Ultimately, it is involved in stimulating transcriptional activation of genes involved in proliferation, differentiation, migration, and viability.

The suppressor of mothers against decapentaplegic (SMAD) pathway is one of the dominant downstream transduction pathways of transforming growth factor-β (TGF-β), of which growth differentiation factor (GDF) and bone morphogenetic protein (BMP) are members (Figure 2) [54,55].

In the canonical pathway, TGF-β binds directly to the extracellular-membrane-bound TGF-β type 2 receptor (TGF-βRII); this ligand–receptor unit recruits and binds to TGF- β type I receptor (TGF-βRI-ALK 4,5,7). The resulting complex phosphorylates and the signal is transmitted into the interior of the cell. This facilitates the binding of SMAD2 and SMAD3 and their subsequent phosphorylation by TGF-βRI kinase activity. Similarly, GDF/BMP binds to TGF-βRII and TGF-βRI (ALK 2,3,6), facilitating the binding and phosphorylation of the SMAD1/5/8 complex [56]. The phosphorylated SMAD2/3 or SMAD1/5/8 complex binds to SMAD4 and is shuttled into the cell nucleus. SMADs accumulate in the nucleus, bind to DNA, interact with various transcription factors to modulate gene expression patterns of target genes, and initiate tenogenic differentiation [57]. SMAD signalling stimulates the expression of Scx and downstream Tnmd, essential factors for the tenogenic differentiation of MSCs [54]. Furthermore, TGF-β/SMAD signalling is essential for the maintenance of tenogenic phenotype of terminally differentiated cells [58]. TGF-β is upregulated during tendon injury and TGF-β/SMAD signalling was identified as a requirement for tenocyte recruitment and functional restoration in neonates [59]. TGF-β signalling enhances the proliferation and migration of TDSCs, promoting tenogenesis [60]. Lastly, TGF-β promotes the synthesis of ECM components, such as collagen, providing a structural framework for developing functional tendons [61]. This pathway does not function in isolation as TGF-β-RII/RI signalling facilitates the activation of non-SMAD (non-canonical) signalling pathways, such as MAPK/ERK, JNK, PI3K/Akt, YAP, and Rho-like GTPase pathways, that function in transcription regulation.

### 2.2. Non-SMAD Signalling

Non-SMAD signalling typically has three physiological effects: the modulation of SMAD protein activity by non-SMAD proteins, crosstalks between SMAD and non-SMAD signalling, and the SMAD activation of non-SMAD pathways and synergism [62].

Apart from the conical pathway, TGF-β_1&2_ stimulation activates the Act-mTOR pathway and mediates tenogenic differentiation (Col I and Tnmd) in the absence of SMAD3 [63,64]. BMP-12 stimulates the translocation of YAP (Yes-associated protein), a transcription co-factor, from the cytoplasm to the nucleus. YAP’s presence serves as a regulator for tenogenic gene expression (Scx, TNC, and Tnmd) [65]. BMP-14 stimulation upregulated sirtuin-1 expression and JNK phosphorylation in a study, triggering the deacetylation of PPARγ and increased expression of Scx [66]. Furthermore, BMP-14 stimulates tenogenic differentiation and Tnmd expression by the phosphorylation of p38 [67]. ERK signalling is mediated by the sequential phosphorylation and activation of RAS, RAF, MEK1/2, and ERK1/2. Ultimately, ERK translocates to the nucleus and regulates tenocyte proliferation and differentiation [68]. CTGF exposure stimulates the phosphorylation of ERK1/2 and FAK, facilitating tenogenic differentiation (Scx, Tnmd, and Col I) and proliferation [69]. The activation of the PI3K/Akt (IGF-1) and MAPK (IGF-1, bFGF, and PDGF) pathways stimulates DNA synthesis and protein synthesis with downstream effects of proliferation, cell survival, cytoskeletal remodelling, and motility [68].

### 2.3. Topography-Based Signalling

Physical cues (e.g., topography and mechanical stretching) from the surrounding microenvironment guide stem cell differentiation into various lineages [70]. Integrin proteins form clusters on the extracellular membrane known as focal adhesions, binding the ECM to the cytoskeleton of the cell. This interaction enables cells to sense the ECM environment and adjust cellular responses (stress fibre formation, contraction, and shape) accordingly [71,72,73]. The Rho/ROCK pathway is the main signal transduction pathway mediating stretch and topography-induced tenogenesis [74]. In a study, focal adhesion kinase (FAK) and tenogenic specific genes were upregulated in TDSCs cultured on micro-surfaces compared to smooth surfaces during integrin binding. The addition of FAK inhibitors negated the tenogenic differentiation of MSCs [75]. Similarly, mechanical stretching increased FAK phosphorylation, actin fibre density, cell elongation, cell alignment, and the tenogenic differentiation of BMSCs. These effects were negated by Rho/ROCK, FAK, and actin polymerisation inhibitors [76]. Disruption to the Rho/ROCK pathway and cytoskeleton resulted in a loss of tenogenic differentiation markers and morphology, suggesting that the Rho/ROCK pathway has a direct effect on tenogenic differentiation [73]. The Rho/ROCK pathway also plays a modulatory role by exerting either a positive or negative influence on TGF-B/SMAD signalling through the regulation of different SMAD phosphorylation sites [74]. The inhibition of TGF signalling suppresses both integrin and Rho/ROCK expression, highlighting the significant interplay and interdependence between these two pathways [77,78]. Furthermore, in a study, the endogenous expression of TGF-β_1_ was upregulated in fibroblasts forced to assume an elongated shape due to surface topography; the elongated shape also made cells more sensitive to TGF-β_1_ [79]. YAP (Yes-associated protein) and TAZ (transcription coactivator with PDZ-binding motif) is another pivotal mechano-sensing pathway, guiding tenogenic differentiation. Similarly, it also shares a crosstalk with TGF/SMAD signalling by facilitating the nuclear localisation of SMAD2/3 [80]. Furthermore, increases in ECM stiffness both activate the Rho-ROCK pathway and lead to F-actin stress fibre formation and YAP translocation [71]. YAP localises in the nucleus in stem cells, regulating tenogenic differentiation [65,81]. Additionally, biomaterials with aligned nanofibers, micro-grooved surfaces, or biomaterials with integrin adhesion sites enhance cytoskeletal tension, facilitating nuclear YAP/TAZ translocation [82,83,84].

## 3. Biomaterial Characteristics to Consider When Designing Tenogenic Differentiation Protocols

### 3.1. Introduction to Biomaterials

**Figure 3:**
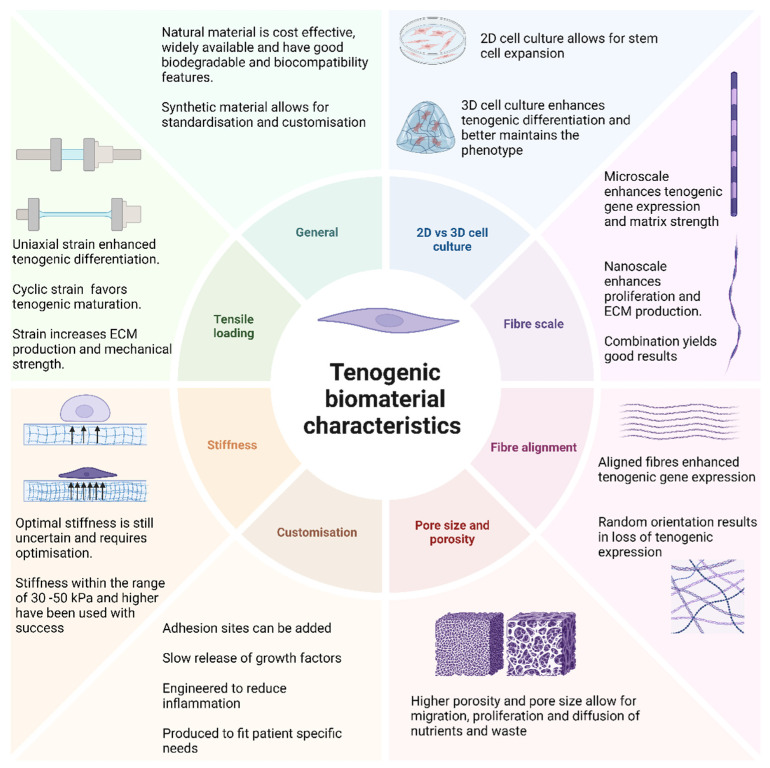
Ideal biomaterial characteristics to enhance tenogenic differentiation. Biomaterial properties have been identified to significantly impact the efficacy of tenogenic differentiation. The ideal biomaterial for tenogenic differentiation is envisioned to have a 3D structure to enhance cellular interaction, utilize a combination of aligned micro- and nanoscale fibres similar to native tendon tissue, and have a higher porosity for cellular migration and diffusion of nutrients; the construct should have a stiffness in the range of 30–150 kPa to enhance the tenogenic phenotype and undergo uniaxial, cyclic strain to facilitate in vivo usage and maturation of the regenerating tissue.

Biomaterials are defined as non-viable materials designed to interact with biological systems and their ideal characteristics will vary depending on the tissue of interest (Figure 3) [85]. These structures mimic the ECMs of tissues, such as tendons, to provide cells with an environment conducive to attachment, migration, proliferation, and differentiation; provide mechanical support to the surrounding tissue; and guide the formation of the regenerating tissue [52]. Tenogenic biomaterials can be classified as natural or synthetic. Natural biomaterials can be protein-based, such as collagen, fibrin, and silk, or polysaccharide-based, such as hyaluronic acid, agarose, chitosan, and alginate [61]. The advantages of using natural biomaterials include cost-effectiveness as these materials are widely available, biocompatible, and biodegradable [61]. However, these benefits are contrasted by several disadvantages such as variations in batch composition, the risk of impurities, rapid degradability, and low mechanical strength [86]. One of the most commonly utilised natural tenogenic biomaterials is collagen as it forms the main structural component of native tendon tissue. Synthetic biomaterials (polystyrene, poly-L-lactic acid, polylactic acid, and polyglycolic acid) are highly reproducible and allow for customisation. However, they tend to have a reduced capacity for biocompatibility, biodegradability, and cytotoxic breakdown products and offer no cell attachment sites [61,87].

### 3.2. 2D vs. 3D Cell Culture

Historically, cells have been cultured in a monolayer within a 2D (two-dimensional) environment. This platform offers several benefits such as simplicity, cost-effectiveness, and ease of observation and the manipulation of cells [88]. However, these advantages are contrasted by several significant limitations such as a lack of biomechanical cues, altered cell behaviour, reduced cellular interaction, and limited physiological relevance [89]. This is evident in early tenogenic differentiation protocols, where tenogenic gene expression was not maintained and phenotype drift during serial passages in 2D cultures were observed [48,90], thus necessitating the need for a paradigm shift towards 3D (three-dimensional) cell cultures, specifically for tenogenic differentiation. This revolutionary approach offers more physiological relevance by mimicking native tissue environments, enhancing differentiation, and allowing for increased cell-to-cell and cell-to-matrix interactions [91]. A three-dimensional (3D) cell culture enhanced the expression of tenogenic genes (Scx, Col I, Col III, and TNC) compared to 2D cultures in a study [74,92]. Furthermore, using a 2D culture allowed for the multilineage differentiation of TDSCs, but when contrasted with a 3D culture, tenogenic differentiation was favoured and other lineages inhibited [93]. However, 2D cultures might be outdated but not forgotten. Cellular proliferation rates are faster in 2D culture vs. 3D culture methods due to less resistance to cellular movement [94,95]. Two-dimensional cultures might still play a role in modern tenogenic differentiation protocols by serving as part of an early stem cell expansion step within a larger 3D differentiation protocol. Therefore, 2D and 3D cell culture methods are complimentary processes rather than being mutually exclusive for tissue regeneration applications.

### 3.3. Topography

Topography refers to the surface characteristics of biomaterials and includes scale, fibre alignment, pore size, porosity, and adhesion sites. These characteristics confer bioactive properties (adhesion, viability, proliferation, differentiation, and phenotype maintenance) to materials used in regenerative medicine [96].

The scale of the fibres used in the production of biomaterials can influence tenogenic differentiation and the mechanical properties (porosity and strength) of the materials. Microscale fibres are comparable in size to tendon fascicles; their larger diameter assists in cell spreading and enhances the expression of tenogenic differentiation genes, as well as increasing the mechanical strength and stiffness of the material [97,98]. Nanoscale fibres are smaller and comparable to collagen fibrils; their smaller diameter assists with proliferation and ECM production but reduces the mechanical strength of the material [97]. Fibre size and stiffness affect the porosity and pore size of the material. Higher porosity and larger pores allow for better cell migration and proliferation, and support neovascularisation and the diffusion of nutrients and waste, but reduce the mechanical strength of the material [99,100].

Both microscale and nanoscale topography can induce tenogenesis. However, a combination of microscale and nanoscale fibres may offer the best of both worlds by promoting tenogenic differentiation and ECM deposition while maintaining the mechanical properties of the material [101]. Enhanced tenogenic differentiation and cell infiltration was observed when using multiscale fibres in a study [102]. Fibre alignment plays a critical role in tenogenic differentiation as it directly influences cytoskeletal organisation and ECM deposition through the activation of mechanosensitive pathways like the Rho/ROCK and YAP/TAZ pathways. Aligned fibres promote cellular elongation and enhance tenogenic differentiation while randomly orientated fibres reduce the expression of tenogenic genes and promote cellular proliferation [103,104].

Researchers were able to stimulate tenogenesis in ADSCs seeded on tenocyte imprints [105]. Three-dimensional surface topography can drive the tenogenic differentiation of MSCs in the absence of biological or chemical stimulation [73]. Furthermore, tendon-imprinted surface topography maintains the tenogenic phenotype of seeded tenocytes in cultures [106]. The current working theory suggests that biomaterial topography controls the shape of seeded cells, influencing their differentiation [107]. Specifically, micro- and nanoscale parallelly aligned topography favour tenogenic differentiation by inducing an elongated cell shape with the parallel orientation of the seeded MSCs. This configuration mimics the natural tendon environment by simulating the longitudinally aligned collagen fibres associated with tendon tissue, enhancing tenogenic differentiation [108].

### 3.4. Stiffness

Material stiffness has been found to significantly regulate cellular processes such as migration, proliferation, and differentiation [109]. However, biomaterials currently used vary widely in stiffness, affecting tenocyte differentiation efficiency. High stiffness favours osteogenic differentiation while soft stiffness may not provide adequate mechanical cues for tenogenic commitment [110]. The optimal stiffness and stiffness nomenclature (low, medium, and high stiffness) for enhanced tenogenesis is controversial in spite of stiffness being an important factor in tendon engineering, limiting the development of a universal differentiation strategy. In a study, TDSCs demonstrated the increased expression of tenogenic genes using a low stiffness of 2.34 kPa compared to 5.89 and 20.09 kPa [111]. A stiffness of 30–50 kPa was sufficient to induce the tenogenic differentiation of BMSCs [112]. Similarly, a stiffness of 35 kPa conditioned tendon stromal cells to maintain a tendon-specific transcriptome, possibly mediated by the PI3K-Akt signalling pathway [113]. A stiffness of 149.53 kPa enhanced the expression of early and late tenogenic expression markers, facilitated adhesion faster compared to stiffer substrates, and promoted the formation of the characteristic spindle-shaped morphology associated with tenocytes [114]. These positive effects were mediated by the inhibition of ROCK. Furthermore, ROCK inhibition enhanced TGF-β_3_ tenogenesis in both 2D and 3D cultures [115]. In the megapascal range, a stiffness of 100 MPa demonstrated superior tenogenic differentiation. However, when the substrate was aligned, 10 MPa demonstrated similar efficiency [110], emphasizing the greater importance of alignment rather than stiffness for tenogenic differentiation. A stiffness of 2.35 ± 0.36 MPa enhanced tenogenic marker expression in TSPCs and promoted the growth and maturation of collagen fibres by increasing the alignment of deposited collagen and larger collagen fibre diameter [116]. Tenocytes can develop over a broad range of stiffness, considering tendon stiffness changes moving from the osteotendinous junction (7.3 ± 0.97 MPa) and tendon proper (2.8 ± 0.87 MPa) to the musculotendinous junction (1.6 ± 0.67 MPa) in vivo [116,117]. However, further studies are required to determine both optimal stiffness for maximal tenogenic gene expression and maintenance as well as factors that synergize stiffness signals.

### 3.5. Tensile Loading

Tendons are mechanosensitive tissues and experience continuous mechanical stimulation in vivo. In a study, a strain of 8% (1 Hz) on human MSCs resulted in aligned cells, a reduction in stemness markers, collagen production, the upregulation of tenogenic genes (Scx, TNC, Col I, Tnmd, DCN, and Col III), and the inhibition of adipogenic, chondrogenic, and osteogenic genes over 72 h. The positive effects were reduced by a strain of 4% [118]. Synergistic effects between TGF-β_3_-induced tenogenic differentiation and strain allowed for lower loading strains to be effective (2%; 0.5 Hz) [119]. Furthermore, it was suggested that strain does not significantly enhance growth-factor-induced tenogenesis. However, it does improve matrix deposition [120]. Uniaxial strain (6%; 0.25 Hz) favoured tenogenic expression in rat TDSCs compared to biaxial strain. Furthermore, uniaxial strain resulted in the phosphorylation of Akt while inhibiting ERK phosphorylation [93]. Static strain can initiate tenogenic differentiation (Scx expression) in the absence of growth factors. However, cyclic strain in required for tenogenic maturation (Tnmd expression) [121]. As previously reviewed, a mechanical strain of 4–8% seems to be optimal for tenogenic differentiation during cyclic strain conditions [122]. Mechanical stimulation has been implicated in regulating collagen organisation, upregulating tenogenic genes, and improving mechanical strength [118,123,124]. Thus, mechanical loading is a strategy that can be used to augment tenogenic matrix maturation post induction.

### 3.6. Customisation

Biomaterial customisation is essential to enhance tendon regeneration. Adhesion sites mediate cell-to-material binding and facilitate cell adherence, the maintenance of cellular morphology, and the integration and migration of cells. Synthetic biomaterials lack these adhesion sites and therefore require modification. Most commonly, short amino acid sequences, from ECM proteins e.g., RGD peptides, have been engineered into biomaterials to promote cell adhesion [125]. Growth factors have a short half-life in vivo and therefore limited effectivity. However, when incorporated into biomaterials, controlled and sustained release can be achieved, ideally continuous with biomaterial degradation, maintaining a constant tenogenic signal to facilitate tendon repair and ECM production by biomaterial-contained tenogenic cells [126]. A significant challenge within regenerative medicine is the cell death of transplanted cells, associated with mechanical stress and a hostile microenvironment such as inflammation [127]. Regulating the local inflammatory response is crucial as excessive inflammation can impede the healing process and reduce the integration of transplanted cells. Biomaterials can be designed to release nanoparticles containing anti-inflammatory agents, forming an environment conditioned to tissue regeneration and optimising tendon regeneration [128].

### 3.7. Advantages and Limitations of Currently Used Biomaterial Fabrication Techniques

Various fabrication techniques have been utilised to develop tendon biomaterials, but differences in these techniques prevent a standardised protocol from emerging. Three-dimensional bioprinting deposits bioink in a layer-by-layer fashion to produce scaffolds [129]. This technique has high precision but has certain drawbacks limiting its use: currently available bioinks lack strength and dynamic properties and their processes are time-consuming [130]. Electrospinning creates nanofibers using various polymers [131]. This technique provides control over fibre diameter and alignment to mimic native tendon tissue [132]. However, complex manufacturing and difficulty in replicating complex structures and optimisation may limit its use [133]. It is argued that decellularised tendon ECM is best suited due to its mechanical properties and architecture, which is similar native tendon tissue [134]. However, harvesting patient tendons adds to morbidity, and the decellularisation procedure can affect the structural properties of the scaffold; allogenic or xenogenic material poses an immunogenic risk and batch variation may occur [135,136]. Hydrogels provide a 3D network of hydrophilic polymers to generate a scaffold, favouring cell growth and viability, but lack tensile strength and stiffness resembling native tendon tissue [137,138]. Thus, these will need to be hybridised with other fabrication techniques. Further research is required regarding biomaterial fabrication techniques and materials to produce a material that encompasses the identified properties required for tenogenic differentiation. Based on the reviewed literature, a synthetic tendon ECM scaffold, embedded within a hydrogel, would produce the desired carrier for tenogenic differentiation and transplantation. This approach would leverage the 3D structure with associated micro- and nanoscale fibres, optimal stiffness, and the uniaxial loading of the tendon ECM while benefiting from the enhanced growth, viability, and protection against inflammation afforded by the hydrogel. The synthetic nature would protect against batch-to-batch variation, ensuring reliability.

## 4. Growth Factors Stimulating Tenogenic Differentiation

Growth factors (GFs) are agents that bind to cell receptors and drive physiological cellular processes such as adhesion, proliferation, migration, and differentiation [139]. Table 1 summarises various GFs explored for their potential use in tenogenic differentiation protocols. Broadly, tenogenic GFs can be categorised into tenogenic inducers (TGF-β and BMP), which promote tendon differentiation, and matrix inducers (ascorbic acid, CTGF, bFGF, PDGF-BB, and IGF-1), which support the production of ECM, necessary for tendon function and repair. Despite significant progress, a standardised protocol has yet to be established. The primary challenge lies in the considerable variations in study parameters, which hinder reproducibility and consensus on optimal conditions. The main variables include single or combination GFs, different GF concentrations, different timepoints for analysis and protocol duration, sequential or concurrent GF administration, and different cell types utilised. However, standardisation and efficacy optimisation are required since GF treatment will form a key part of tendon engineering strategies. The pre-treatment of MSCs (differentiation) enhances tendon regeneration and GF-directed tenogenesis can synergize with scaffold matrices, which will be required for the implantation of cells into damaged tendons [140,141].

TGF-β is a key inducer in tenogenic differentiation protocols and is involved in all phases of tendon healing. It activates early tenogenic transcription factors (Scx, Mkx, and Egr-1); induces the expression of tenomodulin, a marker of mature tendons; induces the expression of tenogenic matrix genes (THBS-4, TNC, Col I, and Col III); and inhibits DCN [63,64,74,119,120,142,143,144,145,146,147,148]. TGF-β isoforms (TGF-β_1_, TGF-β_2_, and TGF-β_3_) seem to stimulate the expression of similar tenogenic genes. However, no differentiation study has compared the tenogenic efficacy of these three isoforms with one another. TGF-β is essential for inducing and maintaining terminal differentiation (Tnmd expression) of tenocytes [146,147]. However, additional GFs are necessary for matrix production, highlighting the need for distinct an induction and maintenance phases [146]. Furthermore, TGF-β yields better differentiation results when used sequentially with other GFs [149]. Despite its potent tenogenic effects, TGF-β negatively impacts cell viability. Therefore, it should be paired with a GF that stimulates proliferation and viability [150,151].

TGF-β_1_ dominates in the literature as a potent inducer of tenogenic differentiation [53]. Furthermore, it is the first isoform to be upregulated after injury and plays a role in TDSC and tenocyte recruitment and differentiation [59]. However, elevated levels in the late stages of adult tendon healing are frequently associated with fibrotic scar formation and adhesion formation [152]. Recently TGF-β_3_ has been gaining more attention in tenogenic differentiation studies. Its expression gradually increases and demonstrates a stronger Col I and Col III expression ability, but is antagonized by TGF-β_1_ [153]. Furthermore, embryonic healing demonstrates higher levels of TGF-β_3_ and less scar formation compared to high levels of TGF-β_1_ during adult healing [154]. A proposed mechanism was a downregulation of SMAD3 in the conical pathway and upregulation of SMAD7 [155]. TGF-β_2_ has received less attention for tenogenic differentiation studies. This may be attributed to the dominance of TGF-β_1_ in literature. There is an assumption that the isoforms have overlapping functions and TGF-β_1_ and TGF-β_3_ isoforms dominate in healing tendons, suggesting their importance for tissue regeneration [59].

In contrast to tenogenic inducers, several GFs have been identified to promote ECM production. Ascorbic acid is included in most tenogenic media or differentiation protocols to promote Col I and III expression and deposition [143,156]. Similarly, PDGF-BB stimulates Scx expression and collagen deposition [157]. IGF-1 upregulates Scx expression and has a synergistic effect with BMP-12 to increase DCN expression and TGF-β_1_ to increase BGN expression [64,158]. Sequential treatment with FGF-2 (bFGF) (50 ng/mL) has enhanced the TGF-β_1_- and TGF-β_3_-induced tenogenesis of PDLSCs [149]. In contrast, co-treatment of FGF-2 (100 ng/mL) antagonizes the tenogenic inducing effect of TGF-β_1_, reducing maturation (tenomodulin expression) and increasing Scx expression [159]. The major difference between these studies was in sequential treatment vs. co-treatment and GF concentration. Additionally, further studies showed that FGF-2 stimulated TNC and DCN production in prolonged cultures while maintaining DCN expression after tenogenic induction [146,160].

CTGF stimulates the expression of Scx, Tnmd, and Col I while inhibiting osteogenic differentiation and promoting proliferation. CTGF has demonstrated a synergistic increase in Tnmd when paired with TGF-β_1_, indicating its use in ECM production and tendon maturation [69,142]. BMP-12 (GDF-7) can stimulate tenogenic differentiation (Scx, Mkx, DCN, TNC, Col I, and Tnmd expression), but it is not tendon-specific as it can also increase adipogenic and chondrogenic markers [143,147,161,162]. Furthermore, it is less effective compared to TGF-β_3_ and TGF-β_1_ [151,163]. However, BMP-12 demonstrates a synergistic effect with TGF-β to enhance collagen production and metabolic activity [146,147]. BMP-13 and -14 assist with matrix production by increasing Scx and Col I expression and collagen deposition [147,157,164,165,166].

Recently, solubilised tendon ECM has developed traction as a tenogenic growth factor. In essence, it is a GF cocktail naturally enriched with TGF-β_1_, TGF-β_3_, bFGF, and IGF-1 [167,168]. It upregulates various tenogenic genes (Scx, Mkx, Col I, Col III, TNC, and BGN), increases proliferation and metabolic activity, and enhances the expression of integrins and TGF-β receptors [78,167,168,169,170]. However, TGF-β_3_ was more effective than tECM in tenogenic induction in a study, but tECM enhanced TGF-β_3_-induced tenogenesis [168,169]. Although soluble tECM does contain all the required tenogenic GF and can induce tenogenesis, there are several disadvantages for clinical application. It will need to be harvested from animals, causing batch-to-batch variations and a risk for infection, or it will need to be harvested from the patient, further worsening patient morbidity, and tendinopathy-affected tendons should be avoided.

Most studies investigating tenogenic differentiation focus on short-term assessments, typically lasting only 14 days, as summarised in Table 1. This restricted timeframe overlooks the complex and dynamic process of tenocyte maturation, which may require extended, stage-specific GF exposure for stable differentiation and the long-term maintenance of the tenocyte phenotype. Without prolonged or sequential GF delivery, the transient expression of markers like Scx, Tnmd, and collagen type I may not ensure functional, long-term tenocyte stability [171]. Future research should extend differentiation durations beyond 14 days and investigate staged GF administration strategies to evaluate the persistence of tenogenic commitment and enhance protocols for clinical applications.

An efficient tenogenic differentiation protocol should facilitate the tenogenic differentiation of MSCs with the optimal expression and maintenance of tenogenic genes while utilizing the least number of growth factors to reduce cost and facilitate scalability. TGF-β is a potent inducer of tenogenesis in MSCs, but it inhibits DCN required for the maturation of collagen fibres [146]. Therefore, a stepwise differentiation protocol should be utilised to recapitulate innate sequential signalling cascades and allow for a physiologically aligned transition of the stem to a differentiated tenocyte [160,172]. It is envisioned that a tenogenic differentiation protocol should consist of a brief induction phase to commit the MSC to the tenogenic lineage. This should be followed by a maintenance phase to allow for the production and maturation of the ECM. To date, only three studies have attempted a stepwise approach to tenogenic differentiation.

The first study utilised a pre-treatment stage to prime MSCs for tenogenic induction. Sequential pre-treatment with bFGF (FGF-2) (50 ng/mL) enhanced TGF-β_1_ (10 ng/mL)- or TGF-β_3_ (5 ng/mL)-mediated tenogenesis [149]. The second study used TGF-β_1_ and ascorbic acid over a three day period to induce tenogenesis in rat BMSCs, followed by the addition of CTGF, for a further seven days, in the maintenance phase to enhance tenogenic maturation and matrix production [142]. The third study utilised a three-day induction phase using a cocktail of BMP-12, IGF-1, TGF-β_3_, ascorbic acid, and bFGF to induce tenogenesis in human BMSCs, followed by a four-day maintenance phase consisting of bFGF and ascorbic acid to maintain the tenogenic phenotype [146]. However, these studies were conducted in a 2D environment. To the best of our knowledge, no stepwise differentiation has been attempted in 3D cultures. Therefore, further studies are recommended to optimise growth factor combinations, concentrations, and sequential exposure, especially within a 3D environment using human MSCs for more clinically relevant applications.

**Table 1:** Growth factors used to induce tenogenic differentiation in MSCs.

Growth Factor	Cell Type	Concentration	Duration	Effect	Reference
L-ascorbic acid	ASCs	500 µM (AA-2P) 50 µg/mL	5 days and 14 days	Increased Scx expression. Increased extracellular deposition of Col I and III. Optimal concentration was 50 µg/mL.	[126,139]
TGF-β_1_	ASCs, BMSCs, MSCs PDLSCs	10 ng/mL, 20 ng/mL	7 days, 10 days, and 14 days	Upregulates Scx, Mkx, Tnmd, THBS-4, TNC, Col I, and Col III. Inhibits DCN. Activates AKT-mTOR signalling. Optimal concentration was 10 ng/mL.	[62,125,126,132]
TGF-β_2_	BMSCs, MSCs	1 ng/mL, 50 ng/mL	72 h, 14 days, and 21 days	Increased Scx, Tnmd, Col I and TNC expression. Signalling is mediated by AKT-mTOR.	[61,127,128]
TGF-β_3_	ASCs, BMSCs, TMSCs PDLSCs	1 ng/mL, 5 ng/mL, 10 ng/mL, 20 ng/mL	72 h, 7 days, 10 days, and 14 days	Increased Scx, Tnmd, TNC, Col I and Col III expression. Reduces proliferation while enhancing metabolic activity. Inhibits DCN. Signalling is mediated by Smad2/3 phosphorylation and enhanced by ROCK inhibition. Optimal concentration was 5 ng/mL.	[72,113,114,129,130,131,132]
CTGF	ASCs, BMSCs	100 ng/mL	10 days and 14 days	Upregulated Scx, Tnmd, and Col I expression. Increased proliferation. Suppressed osteogenesis. Signalling is mediated through ERK1/2 and FAK pathways. Synergistic effect on TGF-β_1_. Optimal concentration was 100 ng/mL.	[67,125]
bFGF or FGF-2	ASCs, BMSCs, PDLSCs	5 ng/mL, 10 ng/mL, 50 ng/mL, 100 ng/mL	7 days, 10 days, and 28 days	Maintains DCN expression after tenogenic induction. Stimulates TNC and DCN expression in prolonged culture. Antagonizing effect on TGF-β_1,_ should be sequentially separated.	[129,132,142,143]
PDGF-BB	ASCs	20 ng/mL	14 days	Induced Scx expression. Increased collagen deposition and Col I expression.	[140]
BMP-12 or GDF-7	BMSCs	10 ng/mL, 50 ng/mL, 100 ng/mL	5 days and 14 days	Stimulated Scx, Mkx, DCN, TNC, Col I, and Tnmd expression. Enhanced expression of adipocyte markers. Synergizes with TGF-β_3_ to increase collagen production and metabolic activity.	[126,130,144,145]
BMP-13 or GDF-6	ASCs, BMSCs	20 ng/mL	14 days	Induced Scx and Tnmd expression. Increased collagen deposition and Col I and III expression.	[140,147]
BMP-14 or GDF-5	ADSCs, BMSCs	100 ng/mL	10 days, 12 days, and 14 days	Induced Scx expression, Tnmd, and Col I. Synergizes with TGF-β_3_ to increase collagen production and TNC expression. Induces differentiation via p38.	[65,130,148,149]
IGF-1	MSCs	10 ng/mL, 100 ng/mL	10 days and 14 days	Upregulates collagen expression. Has a synergistic effect with BMP-12 (DCN) and TGF-β_1_ (BGN). Activates AKT-mTOR signalling.	[62,141]
Soluble tECM	ASCs and MSCs	50 µg/mL and 1 mg/mL	6, 7, and 14 days	Upregulates Scx, Mkx, Col I, Col III, TNC, and BGN and enhanced expression of integrins and TGF-β receptors. Contains various GFs (TGF-β_1_, TGF-β_3_, bFGF, FGF-2, and IGF-1). TGF-β_3_ was more effective than tECM in tenogenic induction. However, tECM enhanced TGF-β_3_ induced tenogenesis. Increased proliferation and metabolic activity Mechanism of action integrin and TGF-β/SMAD crosstalks. Optimal concentration was 1 mg/mL	[77,150,151,152,153]

## 5. Conclusions, Perspective, and Future Directive

Tendon tissue engineering is the intersection between cells, biomaterials, and bioactive factors to repair, restore, or regenerate aged and damaged tendon tissue. To establish a differentiation protocol, future studies must address the inconsistencies in growth factor combinations, biomaterial properties, culture conditions, mechanical stimulation, and cell types used. We have previously reviewed the advantages and disadvantages of various MSCs and their potential for tendon regeneration with the conclusion of using adipose-derived mesenchymal stem cells [173]. The rationale would be to harvest autologous adipose-derived MSCs from the patient and expand the cells in a 2D culture due to its potential for rapid proliferation rates. Thereafter, the cells would be integrated and differentiated within a biomaterial carrier. The ideal biomaterial should have a 3D structure, consisting of a combination of micro- and nanoscale aligned fibres to mimic the native tendon structure, and have high porosity of a sufficient pore size to ensure the adequate migration of cells and diffusion of nutrients and waste products within the material. Furthermore, the biomaterial should have a stiffness in the range of 30–150 kPa for tenogenic differentiation and should have a customisation feature to allow for stiffness adjustments to reach the stiffness of native tendons (2.8 ± 0.87 MPa) around the time of transplantation. However, optimisation is required due to the large variation in stiffness ranges between various tenogenic differentiation studies and the discrepancy in the nomenclature of “soft, medium and high stiffness” biomaterials. Additionally, the biomaterial should allow for uniaxial cyclic loading to facilitate the functionality of the biomaterial, especially for in vivo applications and to enhance the maturation of the regenerating tissue. The biomaterial should allow for customisation, e.g., the sustained release of GF/s and anti-inflammatory cytokines to modulate the microenvironment in favour of tendon tissue regeneration. Lastly, the selected biomaterial should allow for immediate load bearing post-implantation and degrade at a rate comparable to that of the regenerating tendon tissue [134].

Based on the ideal properties identified, a synthetic decellularized tendon ECM would be an ideal candidate as it already possesses the recommended characteristics [136]. In a study, a decellularized tECM demonstrated a tensile strength higher than that of native tendons, indicating suitable mechanical strength and contributing to its functionality. Furthermore, the decellularized tECM allowed for the sustained release of phytochemical additives, supporting the proof-of-concept [174]. However, the decellularized tECM should not be harvested from the patient to avoid further patient morbidity and also not from animals to avoid immunogenic risks and batch-to-bath variation. It is proposed that as 3D bioprinting advances, it might yield an artificial scaffold similar to a native decellularized tECM, which can be embedded within a hydrogel. This approach would leverage the optimal 3D structure of the tECM, ensuring tenogenic differentiation through the activation of Rho/ROCK and YAP/TAZ mechanosensitive pathways to drive topography-induced differentiation with the added benefit of a physical barrier (hydrogel) to protect the regenerating tissue from mechanical stress (cushioning effect) and the inflammatory background within the transplant site. The hydrogel can be customised to ensure the sustained release of GF/s, to enhance tissue regeneration, and anti-inflammatory cytokines to promote an environment conducive to tissue formation. Furthermore, hydrogel encapsulation promotes an M2 macrophage phenotype, aiding in tissue repair rather than fibrosis [175].

GFs guide the differentiation of MSCs in vitro to ensure the correct tissue is formed in vivo post transplantation. However, there is currently no standardised tenogenic differentiation protocol, with a large variation in the GFs used and their concentrations and durations. Based on the literature reviewed, it is proposed that a tenogenic differentiation protocol should consist of a brief induction phase to commit the MSCs to the tenogenic lineage, followed by a maintenance phase to allow for the production and maturation of ECM components. Three attempts have been made to create a stepwise differentiation procedure to capitalise on sequential differentiation, more aligned to a physiological transition of MSCs to terminally differentiated cells. However, optimisation is required since these studies have been limited to 2D cultures.

In an attempt to formulate a standardised protocol, TGF-β is recommended for the in vitro induction phase: either TGF-β_1_ (10 ng/mL) or TGF-B_3_ (5 ng/mL) [149]. However, TGF-β_3_ would be more suited for in vivo use due to its lower risk of fibrosis [154]. The maintenance phase would require GF/s that promote proliferation or cell viability and ECM production and maturation. This would support the negative effects associated with TGF, such as reduced proliferation and DCN inhibition [143,150,151]. Therefore, it is further recommended to use CTGF (100 ng/mL) and/or BMP-12 (GDF-7) (100 ng/mL) in the maintenance phase as they both show a positive synergistic effect with TGF [142,147]. Lastly, the protocol should include ascorbic acid as a supplement to assist in collagen production [143]. It should be noted that this protocol requires validation and optimisation for application within a 3D environment.

Both biomaterial and GF approaches have shown efficacy in tenogenic differentiation. However, the combined use of physical cues and biochemical stimulation has a complimentary effect and ultimately produces more efficient tenogenic differentiation results [108,141,148]. Future research directives should focus on biomaterial development to replicate the features of decellularized tECMs in a synthetic form to ensure standardisation. Furthermore, tenogenic differentiation protocols need to be standardised and optimised for 3D cell cultures. Additionally, most studies reviewed were conducted within a laboratory-controlled environment. More in vivo studies are required to test the applications within an in vivo environment to facilitate progression to clinical trials.

## Data Availability

No new data were created or analysed in this study.

## Abbreviations

The following abbreviations have been used in this manuscript:

2D	Two-dimensional
3D	Three-dimensional
AA	Ascorbic acid
ADSCs	Adipose-derived mesenchymal stem cells
Akt	Protein kinase B
bFGF	Basic fibroblast growth factor
BMP	Bone morphogenetic protein
BMSCs	Bone-marrow-derived mesenchymal stem cells
CTGF	Connective tissue growth factor
ECM	Extracellular matrix
ERK	Extracellular signal-regulated kinase
FAK	Focal adhesion kinase
FGF-2	Fibroblast growth factor 2
GAGs	Glycosaminoglycans
GDF	Growth differentiation factor
GFs	Growth factors
Hz	Hertz
IFM	Interfascicular matrix
IGF-1	Insulin-like growth factor 1
JNK	c-Jun amino-terminal kinase
MAPK	Mitogen-activated protein kinase
MSCs	Mesenchymal stem cells
PDGF	Platelet derived growth factor
PDLSCs	Periodontal ligament derived stem cells
PDMS	Polydimethylsiloxane
PGs	Proteoglycans
PI3K	Phosphatidylinositol 3-kinase
SMAD	Suppressor of mothers against decapentaplegic
TDSCs	Tendon-derived mesenchymal stem cells
tECM	Tendon extracellular matrix
TGF-β_1_	Transforming growth factor beta 1
TGF-β_2_	Transforming growth factor beta 2
TGF-β_3_	Transforming growth factor beta 3
TGF-βRI	TGF-β type 1 receptor
TGF-βRII	TGF-β type 2 receptor
TMSCs	Tonsil-derived mesenchymal stem cells
TNC	Tenascin-C
Tnmd	Thrombomodulin
YAP	Yes-associated protein
